# Motivated Cue Integration in Alexithymia: Improving Interoception and Emotion Information Processing by Awareness-of-Sensation Techniques

**DOI:** 10.3389/fpsyt.2019.00329

**Published:** 2019-05-10

**Authors:** Idit Shalev

**Affiliations:** Laboratory for Embodiment and Self-Regulation, Department of Psychology, Ariel University, Ariel, Israel

**Keywords:** alexithymia, interoception, embodied cognition, focusing, mindfulness, awareness, motivated cue integration

## Abstract

Recent findings indicate that alexithymia is the result of a multidomain, multidimensional failure of interoception. Whereas much of the literature addresses the cognitive and affective aspects of alexithymia, less is known about the association between the failure of interoception and the process of motivated cue integration. The theory of motivated cue integration integrates high-level control processes with low-level embodied and contextual cues, suggesting that selective attention to internal and contextual cues results in the creation of meaning that, in turn, influences judgment and action generation. Conceptualized as a special case of the cue integration problem, alexithymia may be associated with restricted access to emotional cues, indicating impaired connectivity between low-level embodied cues and top–down goals and values. This problem may also be viewed as a means substitution problem, indicating the individual’s need for alternative multisensory information. Based on this reasoning, interventions that exploit awareness-of-sensation techniques (e.g., mindfulness, experiential approach, focusing) may help to improve the distinction between bodily sensation and interpretation and to create meaning of situational state by substitution of inaccessible affective cues with alternative cues. Accordingly, clinicians and neuropsychologists can help individuals who suffer from alexithymia by training them to use awareness-of-sensation techniques and directing their attention to alternative multisensory cues as well as alternative cognitive configurations (e.g., mental images). Integrating peripheral cues in the moment-by-moment generation of meaning and self-regulation can improve affective judgment through the exchange of inaccessible affective cues with alternative ones.

## Alexithymia as a Marker of Atypical Interoception

Alexithymia is a personality trait characterized by difficulty in experiencing and expressing emotions (1, 2). Viewed as a continuum (3, 4), alexithymia manifests as difficulty in identifying feelings and in distinguishing them from bodily sensations of emotional arousal; difficulty in describing one’s own feelings; and an externally oriented cognitive style, i.e., a focusing of one’s attention externally with little introspection or insight into one’s own feelings (5). Recent findings indicate that alexithymia is the result of a multidomain, multidimensional failure of interoception (6, 7). Garfinkel and Critchley (8) differentiated between the different dimensions of interoception. Accordingly, they defined interoceptive accuracy as the ability to detect signals from within the body and interoceptive sensibility as the ability to report on body states (9). Lastly, interoceptive awareness refers to both the awareness of body states and confidence in the accuracy of interoceptive states [see Ref. (10)]. From the perspective of the dimensional elements of interoception and in light of the results of recent research, the impairment in alexithymia may be associated both with reduced interoceptive accuracy and with poor integration of interoceptive information with ongoing cognition, regardless of the interoceptive signal under consideration (7).

There is evidence that both the symptom commonalities between psychiatric disorders and the symptom heterogeneity within disorders may be based on interoception (11, 12). The link between interoception and alexithymia accounts for symptom intercorrelations, suggesting that interoceptive ability may underpin the p-factor, a first-order umbrella factor that describes the severity of psychopathology and its associated neural dysfunction and that is found by performing confirmatory factor analysis on the co-occurrence of particular symptoms across diagnostic categories (6, 13–15). Accordingly, the failure of interoception found in alexithymia has been shown to affect functions of higher cognition such as learning (16), decision-making (17), emotion processing (18–21), and cognitive control (22).

Following this view, the neural processing in alexithymia is activated mainly on the physiological, motor-expressive level and less in associating the produced information with cognitive and emotional response domains (22, 23). These and similar findings suggest that alexithymics exhibit amplified activity—shown by greater hormonal arousal responses during visceral pain—in brain areas believed to be involved in physical sensation. Likewise, increased activity has been reported in the insula, anterior cingulate cortex, and midbrain (22, 23). These structural deficits reveal themselves in complex social situations, indicating that alexithymia presents with limited subjective awareness of the internal state of the body along with impaired cognitive processing of emotion. Alexithymics thus may be unable to use affective signals as guidance for their behavior. Following this view, affected individuals are characterized by exaggerated concentration on and amplification of the somatic sensations associated with the emotional arousal caused by alexithymia. The misinterpretation of these sensations can foster hypersensitivity to bodily sensations and somatic complaints and can lead to hypochondriasis or somatization disorder (24). Indeed, the failure to cognitively regulate distressing emotions may cause prolonged states of sympathetic nervous system arousal (25) that, in turn, could contribute to the development of certain types of somatic illness, such as functional gastrointestinal disorders (26) and essential hypertension (27).

At the neuropsychological level, findings demonstrate that processing and automatically using high-arousal emotional information to respond to concomitant behavioral demands is difficult in alexithymia (28). Likewise, there are strong indications that alexithymic characteristics are associated with impairments in the controlled processing of facial and lexical emotion stimuli. Research has shown that alexithymic individuals suffer from deficits in the automatic recognition of affective valence and reduced involuntary allocation of attention toward emotional information. Such deficits are associated with difficulty in developing healthy emotional reactions and in understanding emotional stimuli at a conscious or controlled level of processing (29).

These findings help explain the feelings of stress, ambiguity, and indecision reported by individuals who suffer from alexithymia. The difficulty alexithymic people have in learning and in decision-making may exacerbate the levels of stress they feel when confronted with the constantly changing environmental demands of daily life. Likewise, the mismatch between physiological arousal and emotional awareness that manifests in alexithymia may inhibit decision-making in alexithymic individuals (30).

### Theory of Motivated Cue Integration

The theory of motivated cue integration (MCI) (31, 32) explains how individuals integrate goals, embodied cues, and multisensory information to create meaning. On the one hand, active goals influence the feasibility of relevant embodied cues (31). On the other hand, the perceiver’s likelihood of drawing a specific inference may be proportional to the strengths of the associations between the contextual cues and multisensory data encountered by the individual (33). The MCI theory integrates high-level control processes with low-level embodied and contextual cues. Thus, according to MCI, selective attention to internal and contextual cues results in different patterns of organization that, in turn, influence judgment and action generation. From a neurophysiological perspective, MCI explains the interaction between the dorsolateral areas of the brain involved in the control process associated with goals and action generation and the ventromedial areas involved in motivation and value.

In line with control theories, goal systems theory (34) suggests that the individual’s choice of actions is driven by the mental representation of goals that they chronically hold or is elicited by the contextual cues of the given situation [e.g., Ref. (35, 36)]. Goals are defined as cognitive representations of desired end points that affect evaluations, emotions, and behaviors (37). The relations between goals and means are depicted in terms of an interconnected cognitive architecture [see also Ref. (38)], wherein a superordinate goal is connected to lower-level, or subordinate, goals that, in turn, are linked to their own means of attainment (35, 39). There potentially exist several alternative means to the same goal that could substitute for each other. Whereas goal systems theory has demonstrated the role of the control process to address situational demands (34), little is known of the integration between control processes and homeostatic and embodied signals. Some research of the socio-emotional aspects of embodied cognition has demonstrated the association between physical sensation and active goals. For example, the findings of Bargh and Shalev (40) indicated that physical and social warmth are substitutable to address the problem of loneliness. Zhang and Risen (41) found that feeling physically cold motivated people to seek social warmth. Likewise, the results of Fay and Maner (42) indicated that warmth satisfied active affiliative motives. Whereas these findings indicate the association between embodied homeostatic cues and interpretation of psychological situational state [see Refs. (43–45)], little is known about the general process of MCI, especially under conditions of affective cue deficits.

To fill this gap in knowledge, from the perspective of MCI, Shalev (31, 32) suggested that individual differences could be partially explained by the types of integration they make between accessible perceptual cues and their unique cognitive configurations (e.g., multisensory input, emotions, mental images). Individuals’ patterns of structural and motivational constraints influence the way they organize semantic information, which shapes the meaning of the psychological experience. Some representative examples of structural and motivational constraints include the unique associations between goals and means (31), the repeated coupling of sensory signals (46), and the strength of the association between specific bodily sensations and psychological concepts such as the association between homeostatic cues (e.g., temperature, dryness) and psychological concepts (40, 43–45, 47). The cue integration process is influenced by situational demands, past experience, and psychiatric and neuropsychological deficits (e.g., cognitive flexibility) (31).

### Alexithymia and the Theory of Motivated Cue Integration

MCI thus provides a framework within which to understand the problem of interoception and affective information processing in alexithymia. Conceptualized as a special case of the cue integration problem, alexithymia may be caused by individuals’ restricted access to the contribution of emotion to their means for goal attainment and by a lack of connectivity between their low-level embodied cues and their top–down goals and values.

That is because emotions are the instantaneous, moment-to-moment output of a continuous sequence of behavior and evaluations of situational demands that enable rapid mobilization and action initiation (48). Put differently, emotions are self-regulatory responses that people exploit to efficiently coordinate themselves toward goal-directed behavior. As Frijda (49) noted, specific emotions imply specific eliciting stimuli, specific action tendencies, and specific reinforcers. When it functions properly, this system allows one to flexibly adapt to changing environmental demands. Inefficient affective information processing or lack of association between embodied cues and top–down goals, in contrast, leads to affective dysregulation and inaction. When this inefficiency becomes severe, various forms of pathology are said to exist (50).

The theory of MCI suggests that the problem of low access to emotional information be viewed as a means substitution problem (34), indicating the individual’s need to switch to alternative means when goal progress *via* a prior means was thwarted. Accordingly, MCI suggests that selective attention to low-level sensory data and mental images may help fill the gap created by the lack of access to emotion among alexithymic individuals.

The difficulty accessing affective signals that is described for alexithymia dovetails well with the skills that are targeted for development in mindfulness and other awareness-of-sensation techniques.

### Use of Awareness-of-Sensation Techniques to Reduce Alexithymia

Mindfulness and other awareness-of-sensation techniques promote an open and conscious awareness of experience that is achieved by observing and acknowledging subjective experiences. Furthermore, it encourages individuals to develop a qualitative and articulated appreciation of their present experiences (51) that enables one to direct selective attention to peripheral cues and to re-integrate these cues to create meaning. Although initial evidence from a meta-analysis of 17 studies suggests that deficient emotional clarity, a major characteristic of alexithymia, may be enhanced by mindfulness-based interventions, there is a need for additional research (52). Research in the practice of mindfulness termed one’s experience of an openness to sensory data “decentering” (53), which describes how an individual increases his or her awareness of his or her unique motivational constraints (e.g., strength of association, type of mental images, metaphors) and of multiple sporadic multisensory signals. As such, decentering functions to reset the emotional context associated with prior judgment to a state of low arousal grounded in visceral sensations. Based on this reasoning, decentering enables the neural principle of interoceptive recovery or the restoration of suppressed viscerosensory brain circuits after emotional challenge (54). A similar process of attention to the flow of internal cues that was recognized early by the humanistic approach was termed “experiencing” (55), which was defined as the manner in which the individual attends to the continuous flow of sensory data, known as feeling. While Rogers’ conceptualization placed a strong emphasis on how the person’s feelings and constructs combined to contribute to his or her notion of self, the approach of others like Gendlin (56) stressed the perceived shifts in the actual bodily sensations associated with the person’s feelings and the insights that emerged from these changes in bodily experience. In line with this view, the practice of focusing suggests that in attending to bodily sensations, the focuser assigns to them words, mental images, or phrases that express the present sensory experience. These words or images can be tested against the bodily sensations, which will not resonate with words or phrases that do not adequately describe them. Once the focuser has accurately linked sensations with words, new words or images emerge that provide the focuser with new insight into the experienced context. Eventually, there will be a shift in experiencing as the person gains some clarity about his or her emotional state and begins to move forward (57).

Following this reasoning, clinicians and neuropsychologists can help individuals who suffer from alexithymia by coaching them to use awareness-of-sensation techniques. Not only can this promote relaxation or self-acceptance; it can also facilitate interoceptive improvement by better distinction between bodily sensation and psychological interpretation. Likewise, integrating peripheral cues in the moment-by-moment creation of meaning can improve affective judgment through the exchange of inaccessible affective cues with alternative ones. Future research will focus on evaluating the effectiveness of these techniques in improving judgment that results in appropriate action generation in contexts involving emotion. Such investigations will contribute to the state of the art of evidence-based research in the area of moment-by-moment self-regulation (32) and interoceptive awareness and the possible synergy between the two.

## Data Availability Statement

No datasets were generated or analyzed for this study.

## Author Contributions

The author confirms being the sole contributor of this work and has approved it for publication.

## Funding

This research was funded by Ariel University research authority internal grant number RA1900000095 to IS.

## Conflict of Interest Statement

The author declares that the research was conducted in the absence of any commercial or financial relationships that could be construed as a potential conflict of interest.
